# Find the match! A tool for residue-specific analysis of epitopes in Bet v 1-like allergens

**DOI:** 10.1186/2045-7022-4-S2-P9

**Published:** 2014-03-17

**Authors:** Dirk Schiller, Hanna Berkner, Maximilian Hartl, Christian Seutter von Loetzen, Michaela Gubesch, Felix Husslik, Daniela Weigand, Iris Lauer, Andreas Reuter, Jonas Lidholm, Barbara Ballmer-Weber, Stefan Vieths, Paul Rösch, Dirk Schiller

**Affiliations:** 1 Paul-Ehrlich-Institut, Divison of Allergology, Langen, Germany; 2 University of Bayreuth, Department of Biopolymers, Bayreuth, Germany; 3 Thermo Fisher Scientific, ImmunoDiagnostics Division, Uppsala, Sweden; 4 University Hospital, Allergy Unit, Zurich, Switzerland

## Background

Birch pollen-allergic subjects often develop Bet v 1-specific IgE that cross-reacts with homologous food allergens. Bet v 1 and its homologs in pollen and food display exclusively conformational epitopes. We established a system to specifically analyze epitope cross-reactivity of Bet v 1-related allergens. The enzyme norcoclaurine synthase (NCS) from Thalictrum flavum is structurally homologous to Bet v 1 but does not bind IgE reacting with Bet v 1-like allergens. By substituting amino acids in a variant of NCS, the impact of individual residues of Bet v 1-like allergens in IgE binding can be studied.

## Methods

Residues potentially involved in IgE binding of Bet v 1 were inserted into NCS. NCS variants were purified and protein secondary and tertiary structures were evaluated by CD and NMR spectroscopy. Sera of birch pollen allergic patients were analyzed for IgE binding to NCS variants and Bet v 1-like food allergens. Cross-reactivity of patients' IgE was assessed by competitive binding assays using variants of NCS and Bet v 1 as well as Cor a 1, Dau c 1, Api g 1, Pru av 1, and Gly m 4, respectively.

## Results

CD spectra and ^1^H^15^N-HSQC NMR indicated Bet v 1-like structure of NCS variants. sIgE binding increased significantly with the number of amino acids substituted in NCS from ≤ 0.35 kU_A_/L in 94% (65/69) of sera to CAP values up to 17.4 kU_A_/L for NCS_N57/I58E/D60N/V63P/D68K_ (NCS_5x) in 68% (47/69) of sera. IgE interaction of NCS_5x could be competitively inhibited with Bet v 1, Cor a 1, and Gly m 4, whereas strongly reduced inhibition was observed with Bet v 1_N43A/E45S/N47A/K55A_, Api g 1, Pru av 1, and Dau c 1, respectively. Structural analysis of the IgE-binding site of NCS_5x showed high conformational similarity with Bet v 1, Cor a 1 and Gly m 4 and low similarity with Pru av 1, Api g 1, and Dau c 1, respectively. Murine monoclonal antibody BV16 abolished Bet v 1-specific inhibition of IgE binding to NCS_5x indicating an overlap between an IgE and IgG epitope.

## Conclusions

Five residues critical for IgE cross-reactivity in a subgroup of Bet v 1-like allergens were identified. NCS_5x bound IgE that cross-reacted only with Bet v 1-related allergens sharing an epitope structure similar to that of NCS_5x. Thus non-allergenic NCS with its Bet v 1-homologous structure is a powerful system for the residue-specific analysis of epitopes of Bet v 1-related allergens.

